# Extrathoracic airway hyperresponsiveness as a mechanism of post infectious cough: case report

**DOI:** 10.1186/1745-9974-4-7

**Published:** 2008-08-04

**Authors:** Nicole M Ryan, Peter G Gibson

**Affiliations:** 1 School of Medicine and Public Health, The University of Newcastle, Callaghan, NSW 2308, Australia; 2 Hunter Medical Research Institute, Department of Respiratory and Sleep Medicine, John Hunter Hospital, Locked Bag 1, Hunter Region Mail Centre, NSW, 2310, Australia

## Abstract

Post-infectious cough is a common diagnosis in people with chronic cough. However, the specific infectious aetiology and cough mechanisms are seldom identified.

We report a case of chronic cough after *Mycoplasma pneumoniae *lower respiratory tract infection with extrathoracic airway hyperresponsiveness as the cough mechanism. Extrathoracic airway hyperresponsiveness may be a common mechanism in post-infectious cough which may be useful both diagnostically and therapeutically since chronic cough with extrathoracic airway hyperresponsiveness responds to speech pathology treatment.

## Background

Post-infectious cough is a common diagnosis, especially in primary care settings, although a specific infectious aetiology is rarely confirmed. Aside from pertussis, the role of other infectious agents in chronic cough is poorly understood. In specialist clinics chronic cough occurs in association with asthma, rhinitis, gastro-oesophageal reflux (GERD), and ACE inhibitor use [1]. However, even in these settings, a respiratory infection is often reported at the onset of chronic cough. Extrathoracic airway hyperresponsiveness (EAHR) represents variable extrathoracic airflow obstruction following inhalation provocation testing [2-6]. It manifests as a fall in inspiratory airflow during challenge with histamine, exercise, or hypertonic saline. EAHR is a feature of cough due to ACE inhibitor use [2], rhinosinusitis [3,4] and GERD [5], and possibly asthma [6]. The mechanism of post-infectious cough is not known, however, upper airway sensory hyperresponsiveness might be one important mechanism in driving cough in some entities of CC [7] and this current case suggests that EAHR may be a useful objective marker and relevant mechanism in post infectious cough.

## Case presentation

A 60 year old non-smoking male presented to the Emergency Department with a non-productive cough and cold symptoms. For the past week he had been confined to bed and reported severe bodily pain, a troublesome cough and shortness of breath when showering and toileting. His temperature was 38.6°C. Physical examination of the chest was unremarkable and chest radiograph showed increased bronchial markings centrally. Arterial Blood Gas results breathing room air were: pH 7.46, pCO_2 _4.6 kPa, pO_2 _6.9 kPa. He was commenced on oral roxithromycin 150 mg bd, inhaled salbutamol 100 ug 2 puffs qid, and analgesia, and continued pre-existing carbamazepine 300 mg bd for controlled epilepsy (a recent onset condition) and thyroxine 50/100 mcg on alternative days for hypothyroidism which had developed five years prior. He was subsequently changed to oral azithromycin 500 mg, improved and was discharged on day 5. Acute and convalescent serology confirmed recent infection with Mycoplasma pneumoniae (antibody titre 1:1280 (ref range < 1:40).

At a seven week follow-up visit he described persistent cough, inspiratory dyspnoea, voice changes (characteristics common to paradoxical vocal cord movement (PVCM) and EAHR disorders) and fatigue. Hypertonic saline provocation test was requested and conducted 2 months later.

**Figure 1:**
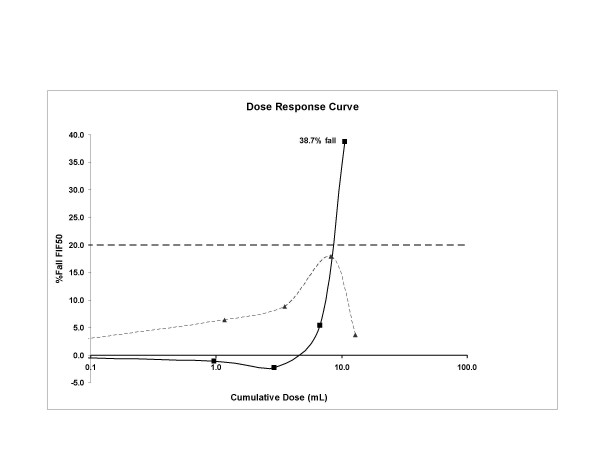
Hypertonic saline provocation dose response curve for FIF_50% _prior to treatment (demonstrating extrathoracic airway hyperresponsiveness) and after treatment. Solid line = pre treatment. Dotted line = post treatment.

Spirometry was FEV_1 _84% predicted, FVC 86% predicted, FEV_1_/FVC 78%; and FIF_50% _5.22 L/sec. Hypertonic (4.5%) saline provocation challenge identified EAHR with attenuation of the inspiratory flow curve. The FIF_50% _decreased by 39% to 3.20 L/s at a cumulative saline dose of 10.59 mL (figure 1, solid line). The fall in FEV_1 _(12%) was within normal limits. A trial of fluticasone/salmeterol and nedocromil sodium was commenced.

The patient's cough and dyspnoea had greatly improved by three months. One year later the cough had resolved completely and an inspiratory/expiratory flow volume curve was normal. There was no EAHR or bronchial hyperresponsiveness after repeat hypertonic saline challenge (figure 1, dotted line), fall in FEV_1 _remained within normal limits (8%) and laryngoscopy showed no posterior chinking during inspiration and no paradoxical vocal cord movement (PVCM).

## Discussion

This case report describes Mycoplasma pneumoniae respiratory tract infection as a cause of persistent cough, occurring in association with EAHR. EAHR was demonstrated by a 39% fall in inspiratory flow during hypertonic saline challenge. The cough resolved as the EAHR resolved. Extrathoracic airway sensory hyperresponsiveness might be an important mechanism in driving cough in some entities of chronic cough (CC) [7]. This case report extends these data to show that transient EAHR can occur with post infectious cough.

It has previously been proposed [8] that some patients with CC sustain vagal injury from respiratory infection and that airway hyperresponsiveness may persist beyond resolution of the acute upper respiratory tract infection (URTI). This hyperresponsiveness could decrease the cough threshold to irritating stimuli resulting in higher susceptibility to chemical or mechanical stimulation of the cough reflex. Transient post-infectious bronchial (intrathoracic) hyperresponsiveness is well recognised [9]. This case report identifies transient EAHR as an additional relevant mechanism associated with post infectious cough.

These observations have implications for the treatment of post infectious cough. There may be a role for inhibition of neuropeptide release, by cromoglycate, nedocromil, or specific neuropeptide antagonists in post infectious cough. Fontana et al [10] evaluated the effects of nedocromil sodium administration on cough threshold in a placebo-controlled study of healthy subjects. They found a significant increase in cough threshold values after nedocromil and an unaffected result after placebo suggesting that nedocromil sodium administration may be useful for treating cough, especially when the use of centrally acting antitussive drugs should be avoided. These agents are also of benefit in ACE Inhibitor cough, which is associated with EAHR. Also, given the similarity between PVCM and EAHR [11], adapting techniques used by speech language therapists that were developed for PVCM maybe of benefit for post infectious cough with EAHR. In PVCM the vocal cords adduct episodically and involuntarily during inspiration. This phenomenon leads to reduced inspiratory airflow associated with signs of stridor and a perception of dyspnoea characterised by the inability to inspire sufficient air [12]. EAHR is thought to be the primary underlying pathophysiology of PVCM [13]. Speech language therapy has been shown to be a successful treatment in chronic persistent cough. Vertigan et al [14] conducted a randomised placebo-controlled trial in 87 patients with CC persisting despite medical treatment. Half of these patients had EAHR and symptoms of PVCM. Patients were randomly assigned to receive either a specifically designed speech pathology intervention or placebo intervention. Participants in the treatment group were found to have a significant reduction in cough with 88% having a successful outcome compared to 14% in the placebo group. In a comprehensive literature review, Gallivan et al [15] presented cases of episodic paroxysmal laryngospasm with definitive diagnosis by videolaryngoscopy of paradoxical vocal cord adduction during inspiration and extrathoracic airway obstruction by attenuation of the inspiratory portion of the flow volume curve. Prior to this, Christopher et al [16] identified 5 patients with a functional disorder of the vocal cords that mimicked attacks of bronchial asthma, that is paroxysms of wheezing and dyspnoea refractory to standard asthma therapy. During episodes of wheezing, the maximal expiratory and inspiratory flow-volume relationship was consistent with variable extrathoracic obstruction. Laryngoscopy confirmed adduction of the true vocal and false vocal cords. While during asymptomatic periods the maximal flow-volume relationship and laryngoscopic examination were normal. Patients were not aware of the vocal-cord dysfunction, which uniformly and dramatically responded to speech language therapy where they were taught to focus attention away from the larynx and the inspiratory phase of breathing during episodes of wheeze and dyspnoea [16]. EAHR may be a useful objective assessment measure to characterise laryngeal dysfunction in chronic cough.

EAHR can be assessed during inhalational provocation challenge. We prefer the use of hypertonic saline to assess EAHR as it is known to provoke neuropeptide release from nonadrenergic-noncholinergic nerves, which are prevalent in the larynx. Inhaled histamine to assess EAHR has been successfully used before [6] where the histamine concentration causing a 25% fall in mid-inspiratory flow was used as the respective threshold of EAHR. It was found that patients presenting with cough as the sole symptom had significantly greater probability of having EAHR. Histamine can however cause oedema of the vocal cords furthering our preference for hypertonic saline stimulus. Methacholine challenge appears to be a less sensitive stimulus for EAHR. This is likely because of its specific action on cholinergic receptors in airway smooth muscle, and unproven action on laryngeal responses. Exercise can also be used to assess EAHR, although quantification of the stimulus may be more difficult.

Our male patient had pre existing hypothyroidism which has been associated with idiopathic chronic cough and airway inflammation [17]. This is unlikely to be the primary cause of cough in the patient as the cough developed after a well-documented *Mycoplasma pneumoniae *lower respiratory tract infection that occurred some 5 years after the onset of hypothyroidism. Further there is a *female *predominance in cases of idiopathic CC and its association with mild chronic lymphocytic airway inflammation [18]. It is however possible that a pre-existing auto-immune lymphocytic bronchitis had a permissive effect on the occurrence of post-Mycoplasma chronic cough. Prospective studies would be helpful in evaluating this possibility.

## Conclusion

Post infectious cough can occur with EAHR. There are opportunities to further investigate the frequency and treatment of EAHR as a mechanism of post-infectious cough with speech pathology.

## Competing interests

The authors declare that they have no competing interests.

## Authors' contributions

NR carried out the flow volume loop and hypertonic saline challenge testing, assisted with laryngoscopy, collected and reviewed data, participated in the design and drafted the manuscript.  PG performed patient physical examination and laryngoscopy, initiated inpatient tests and prescribed medication. PG also participated in the case report design and coordination of the manuscript.

All authors read and approved the final manuscript.

## Consent

Written informed consent was obtained from the patient for publication of this case report. A copy of the written consent is available for review by the Editor-in-Chief of this journal.
